# Preparing future doctors for evidence-based practice: a study on health technology assessment awareness and its predictors in Malaysia

**DOI:** 10.1017/S0266462324000102

**Published:** 2024-02-28

**Authors:** Nur Farhana Mohamad, Zawiah Mansor, Aidalina Mahmud, Izzuna Mudla Mohamed Ghazali, Roza Sarimin

**Affiliations:** 1 Malaysian Health Technology Assessment Section (MaHTAS), Medical Development Division, Ministry of Health Malaysia, Putrajaya, Malaysia; 2 Department of Community Health, Faculty of Medicine and Health Sciences, Universiti Putra Malaysia (UPM), Serdang, Malaysia

**Keywords:** health technology assessment (HTA), awareness, medical student, future doctor, evidence-based practice

## Abstract

**Objectives:**

To determine the level of awareness of health technology assessment (HTA) and its predictors among clinical year medical students in public universities in Klang Valley, Malaysia.

**Methods:**

A cross-sectional study using the stratified random sampling method was conducted among clinical year medical students in four public universities in Klang Valley, Malaysia. Data on the level of awareness of HTA and its associated factors were collected using a self-administered online questionnaire. Descriptive, bivariate, and multivariate analyses were performed using IBM SPSS version 27 to determine the level of awareness of HTA and its predictors.

**Results:**

Majority (69 percent) of participants had a low level of awareness of HTA. The predictors of high-level awareness of HTA were attitude toward HTA (adjusted odds ratio (AOR) = 7.417, 95 percent confidence interval (CI): 3.491, 15.758), peer interaction on HTA (AOR = 0.320, 95 percent CI: 0.115, 0.888), and previous training on HTA (AOR = 4.849, 95 percent CI: 1.096, 21.444).

**Conclusions:**

Most future doctors in public universities exhibit a low awareness of HTA. This study highlights the interplay between attitudes toward HTA, peer interaction, and previous training as influential predictors of HTA awareness. An integrated and comprehensive educational approach is recommended to cultivate a positive attitude and harness the positive aspects of peer interaction while mitigating the potential negative impact of misconceptions. Emphasizing early exposure to HTA concepts through structured programs is crucial for empowering the upcoming generation of healthcare professionals, enabling them to navigate HTA complexities and contribute to evidence-based healthcare practices in Malaysia and beyond.

## Introduction

Rapid advancement of healthcare technology has contributed to rising healthcare costs while offering tremendous improvements in access and outcomes of healthcare services (1;2). In achieving the goal of attaining universal health coverage, countries worldwide have recognized the importance of health technology assessment (HTA) in the decision-making process for health technologies (3). HTA is defined as “a multidisciplinary process that uses explicit methods to determine the value of a health technology at different points in its lifecycle” (4). Its main purpose is to inform decision-making related to health technologies, such as in procurement, funding, appropriate use of health technologies, and for disinvestment in obsolete or ineffective technologies in national, regional, or local healthcare systems (5).

The HTA concept has progressed rapidly in the industrialized nations worldwide as the need for HTA becomes imminent (6). In Malaysia, a formal HTA unit was established in August 1995 under the Medical Development Division, Ministry of Health (MOH) Malaysia (6), which is currently known internationally as the Malaysian Health Technology Assessment Section (MaHTAS). It has been given the mandate to be the center of excellence for informed decision-making for the betterment of the healthcare system by producing transparent, relevant, and accessible HTA reports, as well as fostering collaboration with local and international stakeholders, strengthening HTA capacity in Malaysia, and empowering the consumer (7).

Recognizing the presence of an HTA program or products among stakeholders is acknowledged as the initial level of impact for an HTA organization. This awareness may lead to a change in attitude toward HTA, indicating acceptance of the program and its products. Ultimately, this contributes to influencing decision-making at a later stage (8). Across numerous countries, lack of awareness has been identified as one of the most important barriers and weaknesses in the development, uptake, and implementation of HTA (9–18). The lack of awareness and knowledge has been associated with limitations in the use of HTA in decision-making related to health technologies, from the level of policymaking down to clinical practice (9–18). Misconceptions about HTA were among the top reasons for not using HTA, and particularly for countries with limited resources, the health opportunity cost from misallocating resources in these countries is much higher than in high-income countries (19). Health professionals were unaware of HTA, a tool that can guide difficult choices, especially in balancing organizational and community needs for health technologies. The relatively limited use of HTA due to low awareness may lead to less than well-informed decision-making, which, in turn, may have adverse effects on patients (20).

The World Health Organization 2015 Global Survey on HTA reported that numerous HTA agencies worldwide have recognized the necessity of increasing awareness of HTA among stakeholders, including policymakers, health professionals, educational institutions, and subsequently the public, for the successful implementation of HTA (3). Medical students, particularly the clinical year medical students, are future doctors who have been identified as potential HTA users and that they are the vital target group to instill awareness, basic knowledge, positive attitude, and understanding on the potential contribution of healthcare professionals to the HTA process from the beginning of their medical career to strengthen the implementation of HTA in the future (21).

In Malaysia, the evidence pertaining to awareness of HTA is extremely scarce. Information with regard to exposure or training on HTA among this population is relatively unknown. Ideally, with this information, planning for activities related to creating awareness of HTA among future doctors can be initiated by the universities and HTA organizations to reinforce the use of HTA in Malaysia. However, to date, no single study was found conducted on the awareness of HTA among future doctors in Malaysia or in other countries. Future doctors with low awareness of HTA will potentially fall into the cycle of making unguided decisions related to health technologies when becoming health professionals.

Therefore, this study aimed to determine the level of awareness of HTA and its predictors among clinical year medical students in public universities in Klang Valley, Malaysia, to provide vital information on awareness of HTA in this population, which can be beneficial for academic institutions and HTA agencies in planning for future interventions related to creating awareness on HTA to support the implementation of HTA in Malaysia.

## Methods

This cross-sectional study was conducted in Klang Valley, Malaysia, which is located at the central part of the West Coast of Peninsular Malaysia. The region is dominated by several major city centers that are linked by an extensive form of urban infrastructure (22). The study was conducted over a period of 8 months, from October 2021 until June 2022, involving clinical year medical students in their fourth and final years of medical school from four public universities in Klang Valley, Malaysia. The sample size was estimated based on the two-proportion formula for hypothesis testing by Lemeshow et al. (23) and the sample size required for this study was 358. List of names of all fourth and final year medical students in four public universities in Klang Valley, Malaysia were retrieved from the database in each faculty by the administrative officer of each medical school. Stratified random sampling according to universities and then proportionate to size method were used. Respondents were stratified according to universities and selected according to proportion through simple random sampling by using computer-generated number in Microsoft Excel on the list of clinical year medical students obtained from each faculty. The total number of clinical year medical students required for each medical school was determined by the proportion of the enrollment status in each school. Medical students who were on medical leave and refused to participate were excluded from this study. Another public university in Klang Valley was not included in this study as permission could not be obtained on time.

### Socio-ecological model

In this study, factors associated with awareness of HTA among clinical year medical students are described using a socio-ecological model (SEM) as a framework. The SEM is a well-known and widely accepted framework that has been used extensively to understand individual’s health behaviors better including awareness. Various health promotion programs used the SEM as a guiding framework to get better understanding of health-seeking behaviors, including awareness, as the model considers the complex interplay between individual, relationship, community, and societal factors (24–28). This model describes that an individual’s behavior which is the outcome of interest, is shaped through multilevel factors that include the intrapersonal, interpersonal, institutional, community, and policy levels (29). Using SEM as a framework, factors associated with awareness of HTA among clinical year medical students are identified and grouped into intrapersonal factors, interpersonal factors, institutional factors, and community factors to explore its association with behavior of interest, which is the awareness of HTA. In this study, the dependent variable was the level of awareness of HTA, and the independent variables were:

- Intrapersonal factors: Age, gender, ethnicity, year of study, attitude toward HTA, and personal interest in HTA.
- Interpersonal factors: Peer interaction on HTA, having family members work in health care.
- Institutional factors: Previous training on HTA, previous exposure to HTA, previous training on systematic search, previous training on research methodology, and place of study.
- Community factors: Awareness of online medical research database, awareness of HTA, or Mini-HTA reports.

### Study instrument

Self-administered questionnaire comprised of 26 items in English was used to assess the level of awareness of HTA and its associated factors among the respondents. It consisted of three sections as follows:

**Section A** – Sociodemographic characteristics of respondents (age, gender, ethnicity, year of study, and place of study).

**Section B** – Level of awareness of HTA.

A questionnaire adapted from Noor et al. (30) included items on awareness, which was validated and tested with a Cronbach’s alpha value of 0.81. The level of awareness of HTA was derived from the composite score of five questions consisting of positive and negative responses. Items on awareness were rated using a 5-point Likert scale (30). The responses were summed up and transformed into percentage, which was then categorized according to Bloom’s cut-off point as used in previous studies (30). Scores of >79 percent were used as the cut-off point to define high level of awareness, and scores falling below that threshold were defined as low level of awareness.

**Section C** – Factors associated with level of awareness of HTA.

There were nine items on intrapersonal factors (seven items on attitude toward HTA, one item on personal interest in HTA), two items on interpersonal factors (peer interaction on HTA, having any family members work in health care), four items on institutional factors (previous training on HTA, previous exposure to HTA, previous training on systematic search, previous training on research methodology), and two items on community factors (awareness of any online medical research database, and awareness of any HTA or Mini-HTA reports). For attitude domain, each item was rated using a 5-point Likert scale (30). The responses were summed up and transformed into percentage which was categorized according to Bloom’s cut-off point (30). Scores of >79 percent were used as the cut-off point to define positive attitude, while scores falling below the threshold were categorized as negative attitude.

The questionnaire was assessed by two public health physicians and four HTA experts for clarity, accuracy, language, and relevancy. The content validity ratio for all the questions were calculated. A pretest was done among 30 third-year medical students. A reliability test was conducted, resulting in a Cronbach’s alpha value of 0.720 for the final five items measuring awareness and 0.755 for the final seven items assessing attitude.

This study was registered under the National Medical Research Register (NMRR-22-01032-MI1), and ethical approval was obtained from the Ethics Committee for Research Involving Human Subjects, Universiti Putra Malaysia (JKEUPM). Participation of the study was voluntary, and anonymity of the participants was maintained at all times. Respondents were given patient information sheet, consent, and self-administered questionnaire on Google Forms via link. Consent was obtained prior to answering the online questionnaire. All information was recorded in real time and kept confidential. Alerts and reminders to answer and submit the questionnaire were given to the medical students and student representatives through email notifications twice.

Data collected underwent the process of inputting, cleaning, and analyzing using IBM SPSS (Statistical Package for the Social Sciences) Version 27.0. Descriptive analyses were performed for all variables. Association between level of awareness of HTA and each independent variable were analyzed using chi-square test. The predictors of level of awareness of HTA were determined using binary logistic regression analysis and reported as adjusted odds ratio (AOR) with 95 percent confidence interval (CI).

## Results

### Descriptive analysis

A total of 323 (90 percent) out of 358 students responded to the survey. The distribution of respondents by all factor’s characteristics are shown in Table 1. Median (IQR) age of the respondents was 24 (1). Most respondents were female (69.7 percent) and from Malay ethnicity (74.3 percent). These participants were distributed across the fourth year (51.5 percent) and final year (48.9 percent). Majority of respondents (68.4 percent) had positive attitude toward HTA. For interpersonal factors, most respondents had no personal interest in HTA and no peer interaction on HTA with about 86.4 percent indicated that they have not heard about HTA from peers.

**Table 1.** Distribution of respondents by all factors’ characteristics (*N* = 323)

Factors	Median	IQR	n	%
*Intrapersonal factors*				
Age	24	1		
Between 21 and 24			289	89.5%
Between 25 and 30			34	10.5%
Gender				
Female			225	69.7%
Male			98	30.3%
Ethnicity				
Malay			240	74.3%
Chinese			50	15.5%
Indian			27	8.4%
Other			6	1.9%
Year of study				
Fourth year			165	51.1%
Fifth year			158	48.9%
Attitude toward HTA				
Positive			221	68.4%
Negative			102	31.6%
Personal interest in HTA				
Yes			134	41.5%
No			189	58.5%
*Interpersonal factors*				
Peer interaction on HTA				
Yes			44	13.6%
No			279	86.4%
Family members working in health care				
Yes			143	44.3%
No			180	55.7%
*Institutional factors*				
Previous training on HTA				
Yes			11	3.4%
No			312	96.6%
Previous exposure to HTA				
Yes			37	11.5%
No			286	88.5%
Previous training on systematic search				
Yes			115	35.6%
No			208	64.4%
Previous training on research methodology				
Yes			211	65.3%
No			112	34.7%
*Community factors*				
Awareness of online medical databases				
Yes			295	91.3%
No			28	8.7%
Awareness of HTA and Mini-HTA reports				
Yes			25	7.8%
No			297	92.2%

For institutional factors, large majority of the respondents indicated no previous training on HTA (96.6 percent) and no previous exposure to HTA (88.5 percent). For community factors, most medical students had awareness to online medical research database (91.3 percent) with PubMed and OVID, the most frequent database indicated by the respondents. On the other hand, large majority of the respondents indicated that they were not aware of any HTA or Mini-HTA reports produced by HTA organization in Malaysia (92.2 percent).

### Level of awareness of HTA

The majority of respondents, comprising 69 percent (*n* = 223), demonstrated a low level of awareness, while 31 percent (*n* = 100) exhibited a high level of awareness regarding HTA. Table 2 provides a detailed distribution of responses among the respondents for five statements assessing their awareness of HTA. The analysis of these statements underscores the limited understanding among respondents regarding the function, process, and challenges associated with HTA, as evidenced by a lower percentage of expected answers. The breakdown of responses for each statement further emphasizes specific areas of concern. For instance, most respondents expressed agreement (70.5 percent) that HTA involves critically appraising research findings for clinical decisions. However, a notable percentage (27.6 percent) remained neutral, suggesting a lack of strong conviction or clarity on this fundamental aspect of HTA. A significant proportion of respondents (40.5 percent) either strongly agreed or agreed that HTA does not adequately consider organizational, ethical, and legal aspects. This indicates a potential misconception or lack of awareness regarding the comprehensive nature of HTA.

**Table 2.** Distribution of responses on statements of awareness of HTA among clinical year medical students (*N* = 323)

Awareness statements	Strongly agree *n* (%)	Agree *n* (%)	Neutral *n* (%)	Disagree *n* (%)	Strongly disagree *n* (%)
HTA involves the process of critically appraising research findings as the basis for clinical decisions.	46	182	89	4	2
	(14.2%)	(56.3%)	(27.6%)	(1.2%)	(0.6%)
HTA focuses on the best current available research without considering organizational issues, ethical and legal aspects.	25	106	104	68	20
	(7.7%)	(32.8%)	(32.2%)	(21.1%)	(6.2%)
HTA can be used in situations where there is doubt about any aspect of clinical management.	55	175	84	4	5
	(17%)	(54.2%)	(26.0%)	(1.2%)	(1.5%)
Improving access to summaries of evidence in HTA report is appropriate to encourage evidence-based practice.	63	169	83	5	3
	(19.5%)	(52.3%)	(25.7%)	(1.5%)	(0.9%)
Difficulty in understanding statistical terms is the setback in understanding HTA.	66	137	105	10	5
	(20.4%)	(42.4%)	(32.5%)	(3.1%)	(1.5%)

### Association between intrapersonal factors, interpersonal factors, institutional factors, community factors, and level of awareness of HTA

Results on association between various factors and level of awareness of HTA are presented in Table 3.

**Table 3.** Association between intrapersonal factors, interpersonal factors, institutional factors, community factors, and level of HTA awareness (*N* = 323)

Variable	Low HTA awareness *n* (%)	High HTA awareness *n* (%)	*χ*^2^ statistics (*df*)	*p*-Value
*Intrapersonal factors*				
Age			1.912(1)	.167
21–24 years old	196 (67.8%)	93 (32.3%)		
25–30 years old	27 (79.4%)	7 (20.6%)		
Gender			1.291(1)	.256
Male	72 (73.5%)	26 (26.5%)		
Female	151 (67.1%)	74 (32.9%)		
Ethnicity			4.280(3)	.233
Malay	161 (67.1%)	79 (32.9%)		
Chinese	40 (80.0%)	10 (20.0%)		
Indian	19 (70.4%)	8 (29.6%)		
Others	3 (50.0%)	3 (50.0%)		
Year of study			0.213(1)	.645
Fourth year	112 (67.9%)	53 (32.1%)		
Final year	111 (70.3%)	47 (29.7%)		
Attitude toward HTA			31.216(1)	<.001*
Positive	131 (59.3%)	90 (40.7%)		
Negative	92 (90.2%)	10 (9.8%)		
Personal interest in HTA			0.132 (1)	.717
Yes	129 (68.3%)	60 (31.7%)		
No	94 (70.1%)	40 (29.9%)		
*Interpersonal factors*				
Peer interaction on HTA			0.324(1)	.569
Yes	32(72.7%)	12 (27.3%)		
No	191(79.4%)	88 (31.5%)		
Having family members work in health care			0.004(1)	.947
Yes	99 (69.2%)	44 (30.8%)		
No	124(68.9%)	56 (31.1%)		
*Institutional factors*				
Previous HTA training			2.964 (1)	.085
Yes	5 (45.5%)	6 (54.5%)		
No	218(69.9%)	94(30.1%)		
Previous HTA exposure			2.950 (1)	.086
Yes	21 (56.8%)	16 (43.2%)		
No	202 (70.6%)	84 (29.4%)		
Systematic search training			2.585 (1)	.108
Yes	73 (63.5%)	42 (36.5%)		
No	150 (72.1%)	58 (27.9%)		
Research methodology training			0.458 (1)	.499
Yes	143 (67.8%)	68 (32.2%)		
No	80 (71.4%)	32 (28.6%)		
Place of study			2.582(3)	.461
A	58 (76.3%)	18 (23.7%)		
B	44 (67.7%)	21 (32.3%)		
C	24 (68.6%)	11 (31.4%)		
D	97 (66.0%)	50 (34.0%)		
*Community factors*				
Awareness of online medical research database			1.303 (1)	.254
Yes	201 (68.1%)	94 (31.9%)		
No	22 (78.6%)	6 (21.4%)		
Awareness of HTA or Mini-HTA report			0.351 (1)	.553
Yes	16 (64.0%)	9 (36.0%)		
No	207 (69.7%)	90 (30.3%)		

* Significant *p*-value at <.05; chi-square test.

### Intrapersonal factors and level of awareness of HTA

Only attitude toward HTA was found significantly associated with level of awareness of HTA (*p* < .001). Medical students who had positive attitude toward HTA had statistically significantly higher level of HTA awareness compared to those who had negative attitude toward HTA.

### Interpersonal factors and level of awareness of HTA

No significant association found between peer interaction on HTA and having family members work in health care with level of awareness of HTA.

### Institutional factors and level of awareness of HTA

The findings showed that most respondents with a low level of awareness of HTA had no previous training on HTA and no previous exposure to HTA compared to those who had a high level of awareness of HTA. However, the difference was not statistically significant (*p* > .05). Similar findings were also noted for the association between other factors, including previous training on systematic search (*p* = .108), previous training on research methodology (*p* = .499), and place of study (*p* = .461) with level of awareness of HTA.

### Community factors and level of awareness of HTA

No significant association found between awareness to online medical research databases and awareness of HTA or Mini-HTA report with level of awareness of HTA (*p* > .05).

### Predictors for awareness level of HTA among clinical year medical students

Table 4 displays the predictors for the awareness level of HTA among clinical year medical students in the final model. In this model, predictors for a high level of HTA awareness among future doctors in public universities in Klang Valley, Malaysia were identified as follows: attitude toward HTA (AOR = 7.417, 95 percent CI: 3.491, 15.758); peer interaction on HTA (AOR = 0.320, 95 percent CI: 0.115, 0.888); and previous training on HTA (AOR = 4.849, 95 percent CI: 1.096, 21.444). Students with a positive attitude toward HTA had 7.42 times higher odds of having a high level of HTA awareness compared to those with a negative attitude. Additionally, students who had peer interaction on HTA were 32 percent less likely to have a high level of HTA awareness compared to those who did not, while students with previous training on HTA had 4.85 times higher odds of having a high level of HTA awareness compared to those without such training.

**Table 4.** Predictors for level of awareness of HTA (*N* = 323)

Multiple logistic regression				
Variable	Coefficient	Adjusted OR	*p*-Value	95% CI for odds ratio
Attitude toward HTA				
Negative (Ref)				
Positive	2.004	7.417	<.001**	3.491, 15.758
Peer interaction				
No (Ref)				
Yes	−1.139	0.320	.029*	0.115, 0.888
Previous training on HTA				
No (Ref)				
Yes	1.579	4.849	.037*	1.096, 21.444

* Significance level *p* < .05.

** Significance level *p* < .01.

## Discussion

From this study, it was evident that the level of awareness of HTA among future doctors in public universities in Klang Valley, Malaysia, was low. In contrast, unpublished data from a 2019 cross-sectional study conducted by the MOH Malaysia revealed that about 59.7 percent of healthcare decision-makers were aware of HTA (31). This disparity may be attributed to the different environments of the study settings. The exposure to HTA is higher, and knowledge about HTA is more accessible for healthcare professionals in the MOH compared to medical students. Moreover, an international cross-sectional study in Bulgaria assessed HTA awareness among various stakeholders, reporting an overall percentage of 19.62 percent who were not aware of HTA, with approximately 22 percent of physicians and 15 percent of experts in Bulgaria lacking awareness (32). The study’s focus on a mixed population may have contributed to the observed difference in awareness levels compared to clinical year medical students. Healthcare professionals, particularly decision-makers and experts, likely benefit from prior training and multiple exposures to HTA throughout their careers, contributing to their higher awareness levels compared to clinical year medical students. Low awareness of HTA among clinical year medical students raises concerns about their readiness to make evidence-based decisions in their future practice. For example, when evaluating new medical technologies or drugs, a doctor with limited awareness of HTA may be more susceptible to relying on biased information provided by pharmaceutical companies or personal anecdotes. This reliance could potentially lead to suboptimal treatment choices for patients. Furthermore, low awareness of HTA may result in the improper use of health technologies, even when they are not recommended or deemed unsafe by HTA. In addition, the consequences of low HTA awareness among healthcare professionals may manifest as a failure to request HTA assessments prior to making decisions related to health technologies or an underutilization of HTA reports (31). These issues can contribute to uninformed policy decisions, potentially leading to the misallocation of funds.

The attitude of clinical year medical students toward HTA plays a crucial role in predicting their awareness levels. A positive attitude reflects an open and receptive mindset toward the concept of HTA, making students more likely to seek information and engage with the topic. Given the limited availability of data on HTA awareness, we resorted to drawing parallels with evidence-based medicine (EBM) because of the interconnected nature of EBM and HTA, with overlapping definitions (33). In the comparison between attitudes toward HTA and EBM, a positive attitude was found to be associated with heightened awareness of EBM, familiarity with online medical research databases, and a greater interest in EBM training (34, 35). Students with a positive attitude may be more inclined to proactively engage with HTA concepts, seek out resources, and view HTA as a valuable tool in their future medical careers. This proactive engagement contributes to higher levels of awareness. These findings underscore the pressing need to implement strategies aimed at cultivating positive attitudes toward HTA among medical students. Promotional activities within medical schools that highlight the value and real-world applications of HTA can foster such attitudes. Furthermore, students can benefit from interactive workshops and simulations that allow them to actively engage with HTA concepts, making the subject more relatable and enjoyable and cultivating positive attitudes toward HTA. Additionally, providing access to user-friendly resources and facilitating peer interactions related to HTA can further encourage proactive engagement and contribute to higher levels of awareness among future healthcare professionals.

Peer interaction, where students learn about HTA from their peers, is identified as another predictor of awareness. This finding suggests that the influence of peers can either enhance or hinder awareness levels. For instance, if students engage in discussions with peers who have a strong understanding of HTA, it can positively impact their awareness levels. Conversely, misinformation or misconceptions from peers may lead to reduced awareness, as observed in this study. This might be attributed to the generally low exposure to HTA among future doctors, emphasizing the critical need for accurate peer-to-peer education. Misconceptions about HTA were among the top reasons for health professionals not using HTA, particularly for countries with limited resources (19). Addressing this issue is essential to prevent the perpetuation of misconceptions and to foster a supportive peer learning environment. Additional predictor, the history of previous HTA training, is associated with significantly higher awareness levels among clinical year medical students. This aligns with previous research on EBM, emphasizing the positive impact of structured training programs on awareness and knowledge levels (36–38). The findings suggest that students with prior HTA training possess a more comprehensive understanding of HTA methodologies, significance, and real-world applications. This knowledge equips them to identify and address HTA-related opportunities and challenges more effectively in clinical practice.

These findings collectively emphasize the critical role of structured educational programs in fostering HTA awareness among future doctors. Early exposure to HTA concepts in these programs serves multiple purposes: fostering a positive attitude toward HTA, preventing misconceptions, and encouraging peer interaction. This highlights the integrated approach needed to instill foundational knowledge and cultivate a supportive learning environment through peer engagement. These programs should include introductory workshops covering fundamental HTA concepts, significance in healthcare decision-making, and practical applications. Additionally, integrating HTA modules into the medical curriculum during clinical years is essential, ensuring coverage in relevant courses such as health policy and public health. Case-based learning, involving modules exposing students to real-world HTA applications, is crucial. Encouraging analysis and discussion of cases where HTA plays a pivotal role contributes to practical understanding. Interactive training sessions, such as seminars and expert talks, featuring professionals in HTA, provide opportunities for students to engage, ask questions, and gain practical insights. Additionally, collaborative awareness campaigns can be organized by HTA agencies and academic institutions, targeting both students and faculty through seminars, webinars, and talks to introduce the significance of HTA in healthcare decision-making. For example, HTA agencies can partner with medical universities to conduct an annual HTA awareness week, during which HTA experts deliver informative talks to students and faculty, emphasizing HTA’s role in evidence-based practice. International experiences can be integrated which can then provide specific policy recommendations for reference in Malaysia. Such early exposure can ultimately enhance awareness within this population.

This pioneering study on HTA awareness among clinical year medical students in Malaysia offers foundational insights that can guide the development of targeted programs. This research not only aids local academic institutions and HTA agencies in planning awareness initiatives for future doctors but also holds broader implications for international HTA organizations. The findings serve as a catalyst for policymakers and HTA agencies to consider introductory sessions on HTA, fostering early awareness and better clinical decision-making. Moving forward, the study’s baseline information can inform researchers globally, encouraging further exploration into HTA awareness. Future research avenues should extend beyond medical students to assess HTA understanding among policymakers, pharmacy departments, and public health departments. Exploring perceived barriers and employing robust study designs will provide a comprehensive understanding, paving the way for the integration of HTA into medical education curricula and healthcare practices. This study has several limitations. It concentrated on clinical year medical students exclusively from public universities in Klang Valley, Malaysia, thereby not accounting for potential variations in results from private institutions. Data collection during the early COVID-19 endemic phase relied on self-administered online questionnaires because of online classes, potentially introducing recall bias. It is important to note that these limitations are inherent in the study design, and given the constraints of time and resources, this study represents the most feasible and comprehensive effort to investigate the awareness levels of HTA among the specified demographic.

## Conclusion

The majority of future doctors in public university exhibit a low awareness of HTA. This study highlights the interplay between attitudes toward HTA, peer interaction, and previous training as influential predictors of HTA awareness. An integrated and comprehensive educational approach is recommended to cultivate a positive attitude and harness the positive aspects of peer interaction while mitigating the potential negative impact of misconceptions. This underscores the importance of early exposure to HTA concepts and the implementation of structured educational programs, a strategic approach vital for preparing future doctors in Malaysia for evidence-based practice and empowering the upcoming generation of healthcare professionals to navigate HTA complexities and contribute to evidence-based healthcare practices in Malaysia and beyond.
